# Empowerment of nurses in qatar: unveiling the relationship between self-esteem, psychological empowerment, and assertiveness

**DOI:** 10.1186/s12912-025-03428-8

**Published:** 2025-07-21

**Authors:** George V. Joy, Atul Deshmukh, Kalpana Singh, Abdulqadir J. Nashwan, Badriya Al-Lenjawi

**Affiliations:** 1 Department of Nursing and Midwifery, Hamad Medical Corporation, Doha, Qatar; 2 DY Patil University, deemed to be University, Navi Mumbai, India; 3 University of Doha for Science & Technology, Doha, Qatar

**Keywords:** Self-Esteem, Psychological empowerment, Assertiveness, Nurses, Workforce well-being

## Abstract

**Background:**

Nurses are vital to healthcare systems, yet their psychological well-being remains underexplored despite its critical impact on professional performance and job satisfaction. Essential attributes such as self-esteem, psychological empowerment, and assertiveness play a crucial role in effective decision-making and advocacy. In Qatar, the National Health Strategy (2024–2030) emphasizes healthcare worker empowerment; however, research on the interplay of these psychological constructs among nurses is scarce.

**Aim:**

This study aims to evaluate the levels of self-esteem, psychological empowerment, and assertiveness among nurses in Qatar and examine their interrelationships, particularly the mediating role of self-esteem between empowerment and assertiveness.

**Methodology:**

A quasi-experimental one-group pre-posttest design was employed, focusing on baseline data from 144 registered nurses across Hamad Medical Corporation (HMC) facilities. Data were collected using validated tools, including the Rosenberg Self-Esteem Scale, Psychological Empowerment Scale, and the Rathus Assertiveness Schedule. Statistical analyses, including structural equation modeling and ANOVA, were conducted to identify relationships and demographic associations.

**Results:**

Findings indicated moderate levels of assertiveness (mean = 67.1 ± 10.9) and empowerment (mean = 51.1 ± 5.9), along with high self-esteem (mean = 27.1 ± 2.9). Empowerment significantly influenced assertiveness (β = 0.207, *p* = 0.009); however, self-esteem did not mediate this relationship. Significant demographic variations in self-esteem were observed, with higher levels reported among early-career nurses and general registered nurses, while assertiveness and empowerment remained consistent across demographic groups.

**Conclusion:**

The study underscores the importance of fostering empowerment, self-esteem, and assertiveness among nurses through targeted interventions such as assertiveness training and supportive organizational policies. Future research should explore longitudinal changes in these psychological dynamics to enhance sustained professional development.

**Clinical trial number:**

Not applicable.

## Introduction

Nurses are the backbone of healthcare systems, providing critical care, ensuring patient safety, and serving as advocates for health promotion and improved patient outcomes. Despite their indispensable role, the psychological well-being of nurses often remains overlooked, even though it significantly impacts their professional performance, job satisfaction, and overall quality of care delivery [1]. Central to effective nursing practice are the qualities of self-esteem, psychological empowerment, and assertiveness. These attributes enable nurses to make informed decisions, maintain professional boundaries, and engage confidently in patient advocacy and teamwork [2]. Conversely, non-assertive behavior and low self-esteem are persistent challenges, exacerbating stress, mental fatigue, and dissatisfaction within the profession [3].

Research highlights that many trained nurses exhibit non-assertive tendencies, including an inability to refuse excessive workloads, resulting in heightened stress and frustration [4]. Assertiveness is a cornerstone of professional autonomy and empowerment, enabling nurses to communicate effectively and navigate complex workplace dynamics [5]. Psychological empowerment plays a crucial role in fostering assertiveness, boosting confidence, and promoting healthier work environments [6]. Nevertheless, few studies have explored the direct relationship between empowerment and assertiveness among nurses, emphasizing the need for targeted interventions to address these gaps [7].

The interplay between self-esteem and assertiveness is especially significant. High self-esteem promotes confidence, effective workplace interactions, and resilience against barriers such as fear, poor self-expression, and a lack of confidence [8]. These barriers, often rooted in low self-esteem, hinder nurses’ ability to assert themselves and diminish their professional effectiveness. For nurses in collectivist cultures, societal norms that discourage assertiveness can further compound these challenges, making it even more critical to address issues of empowerment and self-esteem [9].

In the context of Qatar, the Qatar National Health Strategy (2024–2030) underscores the importance of empowering healthcare workers, particularly nurses, to enhance societal productivity and employee well-being [10]. While the strategy emphasizes empowerment and well-being, there remains limited empirical research on the impact of psychological empowerment and assertiveness among nurses in Qatar. This paucity of localized evidence highlights the urgent need for targeted studies to address the unique challenges faced by the nursing workforce in the region.

By fostering self-esteem, psychological empowerment, and assertiveness, healthcare systems can better support nurses in fulfilling their multifaceted roles. This study is a part of a broader psychosocial wellness intervention aimed at enhancing mental well-being among healthcare professionals. The aim of this study is to assess self-esteem, psychological empowerment, and assertiveness among nurses, and to explore the potential mediating effects among these variables. Specifically, the study seeks to answer the following research question: To what extent are self-esteem, psychological empowerment, and assertiveness interrelated among nurses in Qatar, and does self-esteem mediate the relationship between psychological empowerment and assertiveness?

## Theoretical framework

**Fig. 1:**
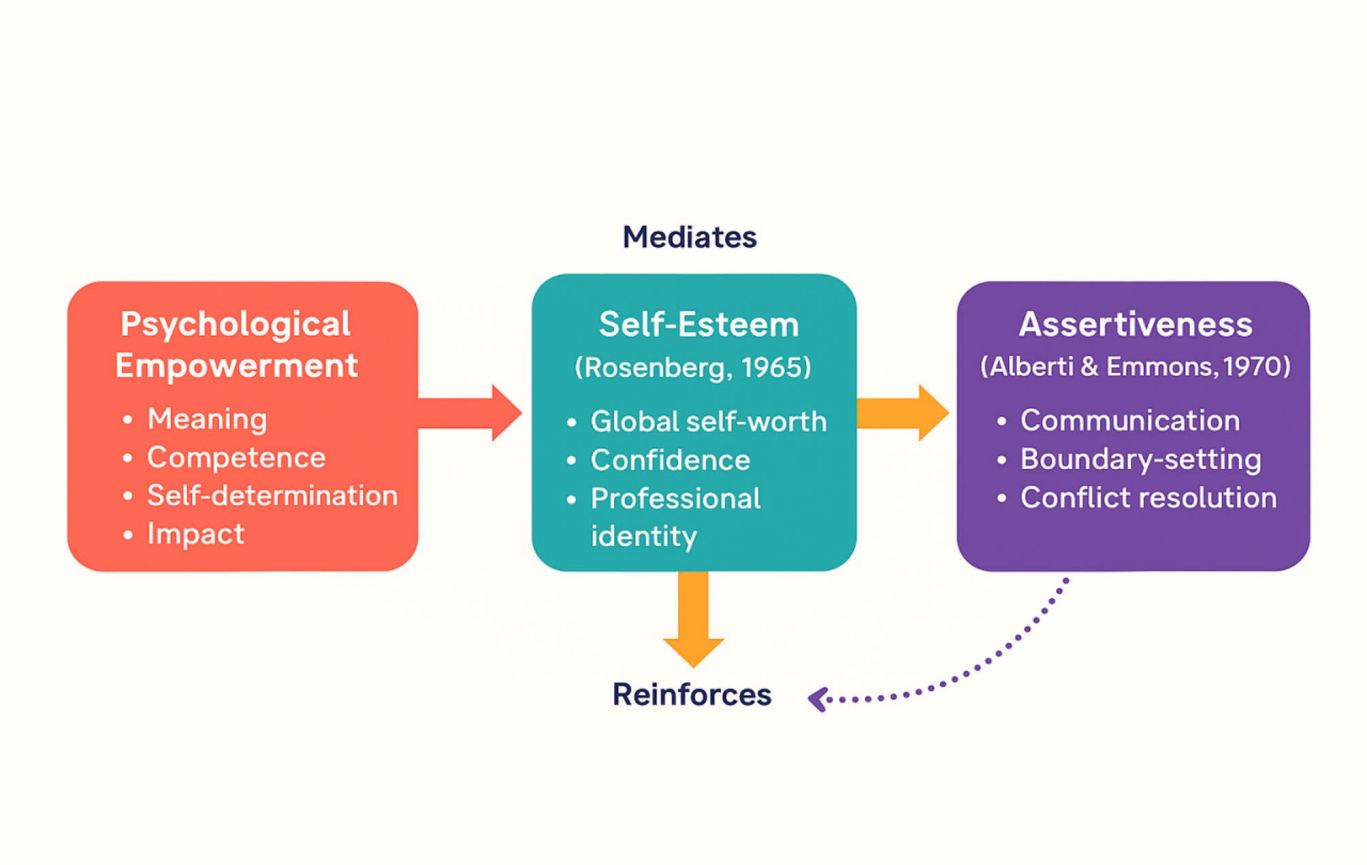
Theoretical framework illustrating self-esteem as a mediator between empowerment and assertiveness in nursing practice

The theoretical framework for this study integrates *Rosenberg’s Self-Esteem Theory* (1965), *Empowerment Theory* (Spreitzer, 1996), and *Assertiveness Theory* (Alberti & Emmons, 1970) to explore the interplay between self-esteem, empowerment, and assertiveness in nursing practice. Rosenberg’s theory defines self-esteem as the individual’s global evaluation of self-worth, which directly influences confidence and resilience in professional settings. For nurses, high self-esteem is critical to navigating workplace challenges and delivering patient care effectively [11]. Spreitzer’s Psychological Empowerment Theory redefines empowerment as an individual-level motivational construct, characterized by four cognitive dimensions: meaning, competence, self-determination, and impact. This framework shifts the focus from structural empowerment to the psychological experience of control and influence within one’s professional role. For nurses, these perceptions are critical for sustaining motivation, enhancing performance, and promoting job satisfaction, particularly in high-pressure healthcare environments [12]. Empowered nurses feel more confident in their roles, leading to improved patient outcomes and increased professional engagement. Assertiveness Theory provides a behavioral lens, highlighting the importance of effective communication, boundary-setting, and self-advocacy in hierarchical healthcare settings. Assertive nurses are better equipped to manage conflicts, advocate for patient needs, and maintain professional boundaries, which mitigates stress and burnout [13]. The interplay between these constructs is dynamic, with self-esteem serving as the mediating mechanism that links empowerment to assertiveness. Self-esteem acts as a mediator, translating the psychological effects of empowerment into confident, assertive behaviors, while assertiveness reinforces and sustains high self-esteem through positive interpersonal and professional interactions [8]. Together, these constructs form a synergistic framework essential for developing resilient, competent, and satisfied nurses. This framework highlights the need for interventions such as assertiveness training, leadership support, and empowerment-focused organizational policies to enhance nursing performance and well-being. Future research can expand on this interaction to address its longitudinal impact on professional outcomes. Figure 1 describes the theoretical framework illustrating self-esteem as a mediator between empowerment and assertiveness.

### Development of psychological intervention based on the frame work

The psychosocial wellness intervention was developed based on the study’s theoretical framework and incorporated techniques and activities grounded in evidence-based practice. It was designed to enhance nurses’ psychological empowerment, self-esteem, and assertiveness by addressing key psychological and social determinants of well-being. The module consisted of three structured sessions, each lasting one hour and delivered over a span of 10 days. The content of the intervention was adapted from validated psychological techniques and practices and was subjected to expert review by a panel of seven professionals, including a psychiatrist, two clinical psychologists, a psychotherapist, a counsellor, and two psychiatric clinical nurse specialists. Based on their feedback, the module was refined prior to implementation. The first session, titled “Manage Your Emotions,” focused on emotional regulation through mental reframing, breathing exercises, emotional freedom tapping, and reflective practice. The second session, “Motivate Yourself,” aimed to strengthen self-esteem and psychological empowerment using mirror-based self-love techniques, positive affirmations, visualization, and emotional empowerment strategies. The third and final session, “Be Assertive,” concentrated on enhancing assertive communication skills through interactive coaching, structured assertiveness exercises, and the application of psychodrama to reinforce learning. Collectively, these sessions were designed to provide nurses with practical tools to improve their emotional well-being, interpersonal confidence, and professional autonomy within clinical settings.

## Methodology

### Study design

The broader study was structured as a quasi-experimental one-group pre-posttest design, commonly employed to assess the effects of interventions in real-world settings where randomization is not feasible. A quasi-experimental design involves observing a single group before and after an intervention to detect any measurable change, offering practical utility in clinical environments like nursing. This design was selected to evaluate a psychosocial wellness intervention aimed at enhancing self-esteem, psychological empowerment, and assertiveness among nurses, without withholding the potential benefits from a control group.

However, the current manuscript reports only the baseline (pre-intervention) data collected prior to the implementation of the intervention. Therefore, while framed within a quasi-experimental methodology, the present analysis is best interpreted as a descriptive correlational study, focusing on cross-sectional relationships between psychological variables. This approach helps establish foundational insights into nurses’ psychological profiles and informs future evaluation of the intervention’s impact using posttest data.

### Participants

The research focused on registered nurses employed across various facilities under Hamad Medical Corporation (HMC), the largest healthcare organization in Qatar. HMC comprises 14 health facilities that collectively address the full spectrum of medical needs for both Qatari citizens and residents. With a workforce of over 10,000 nursing staff, it represents a significant portion of the national healthcare system. To this study, we specifically included nurses who were actively providing care to vulnerable populations within selected institutions, namely the Women’s Wellness and Research Center (WWRC), Qatar Rehabilitation Institute (QRI), Al Wakra Hospital (AWH), and the Mental Health Service (MHS).

Based on the previous study on the assertiveness levels of psychological interventions among nurses, the effect size of pre- and post-intervention was estimated to be 6.89 for a psychological intervention program [14]. The required sample size was calculated to be 131 participants to achieve 90% power with a two-sided α of 0.05. To account for a 10% loss to follow-up, the final sample size was determined to be approximately 144 nurses (pre-intervention and post-intervention). The sample size calculation was performed using STATA 17.0 software.

To ensure a representative sample, proportional stratified random sampling was used. Approximately 7% of the nursing staff from each facility contributed to the sample, with 47 participants from WWRC, 20 from MHS, 21 from QRI, and 56 from AWH. Participants were invited through the Hamad Medical Corporation official email system, along with an information sheet, during a two-month recruitment period. Registered nurses who expressed their willingness to participate during this period were considered for selection.

Eligibility criteria included being a registered nurse with a minimum of one year of clinical experience and proficiency in English. Nurses occupying non-clinical roles or serving as temporary staff were excluded to maintain sample consistency and relevance to the study objectives. This sampling approach ensured equitable representation from each facility and supported the validity of the study’s findings.

### Data collection

A structured questionnaire was used to collect the data. The first part of the questionnaire was the demographic data of the participants. The second part of the questionnaire consisted of three validated scales.

- The Rosenberg Self-Esteem Scale, developed by Morris Rosenberg in 1978, is a widely used 10-item scale for assessing global self-worth. The instrument evaluates both positive and negative perceptions of self-using a 4-point Likert scale, with responses ranging from 1 (Strongly Disagree) to 4 (Strongly Agree**)**. Total scores range from 10 to 40, where higher scores reflect greater self-esteem [15]. The scale has been extensively validated across diverse cultural settings, with reported reliability coefficients ranging from 0.72 to 0.9. Its internal validity is supported with a Cronbach’s alpha of 0.86 [16].
- The Psychological Empowerment Scale, developed by Spreitzer and Gretchen in 1996, is a 12-item instrument designed to measure employees’ psychological empowerment. The scale evaluates empowerment through dimensions such as perceived control, self-perceived competence, and the internalization of organizational goals. Each item is rated on a 7-point Likert scale, ranging from 1 (Very Strongly Disagree) to 7 (Very Strongly Agree), with total scores ranging from 12 to 84. Higher scores indicate greater psychological empowerment [17]. This scale has demonstrated strong psychometric properties, with a Cronbach’s alpha of 0.90 and test-retest reliability estimates of approximately 0.80 [18].
- The Simple Rathus Assertiveness Schedule (SRAS), developed by Spencer Rathus in 1973, is a 19-item instrument used to measure assertiveness levels. Participants rate items on a 6-point Likert scale ranging from 1 (Very Unlike Me) to 6 (Very Much Like Me). Total scores range from 19 to 114, with higher scores indicating greater assertiveness. This tool is also intended for tracking behavioral changes during assertiveness training [19]. The SRAS has demonstrated acceptable reliability (α = 0.81) and construct validity, showing a high correlation (*r* = 0.98, *p* = 0.01) with the original Rathaus assertiveness schedule [20].

### Study procedures

Institutional Review Board (IRB) approval (MRC-01-23-465) was obtained from the Medical Research Centre (MRC) of Hamad Medical Corporation (HMC). An invitation, along with an information sheet detailing the study’s objectives, procedures, and confidentiality measures, was sent to facility nurses through their official emails. Nurses who expressed willingness to participate provided written consent during a face-to-face meeting, as this study was part of an experimental design.

Following the written consent process, data collection was conducted through an online survey via Microsoft Teams, using participants’ official email accounts. The questionnaire was designed to be completed within 15–20 min to minimize respondent burden. To enhance participation rates, reminder emails were sent biweekly during the data collection phase. The anonymized data was securely stored and accessible only to authorized research personnel. Incomplete or invalid responses were excluded from the analysis to maintain data quality and reliability.

### Statistical analysis

Descriptive statistics, including means, standard deviations, frequencies, and proportions, summarized the characteristics of the study sample and the distribution of self-esteem and assertiveness scores. Structural equation modeling (SEM) was used to assess the mediation effect between empowerment, self-esteem, and assertiveness. Prior to conducting SEM, key statistical assumptions were evaluated to ensure the validity of the model. Specifically, the data were tested for multivariate normality using Mardia’s coefficient, and results indicated acceptable levels of skewness and kurtosis. Linearity was assessed through scatterplots, and no violations were observed. Multicollinearity was checked using variance inflation factors (VIF), all of which were below the commonly accepted threshold of 5. Additionally, sample size adequacy was confirmed based on the recommended minimum of 10 observations per estimated parameter. To address potential limitations related to the modest sample size and to improve the reliability of the mediation effect estimates, bootstrapping with 500 replications was used. This non-parametric resampling technique enhanced the robustness of the standard errors and confidence intervals. T-tests and ANOVA were conducted to examine the associations between sociodemographic variables and empowerment, self-esteem, and assertiveness levels. All statistical analyses were conducted using STATA 17.0 software. A p-value of < 0.05 was considered statistically significant, and all tests were two-sided.

## Results

### Demographic and professional characteristics of participants

Descriptive statistics were utilized to summarize the demographic and professional characteristics of the participants. The mean age is 38.1 ± 6.1 years. The majority of participants were female (74.3%) and married (96.5%). Regarding work experience, 42.4% have over 11 years at HMC, 34.7% have 5–10 years, and 22.9% have 1–5 years. Educationally, 81.3% hold a BSN/Diploma, while 18.8% possess a master’s degree or higher. Job positions include 60.8% GRNs, 18.2% charge nurses, 11.9% nurse educators, and 9.1% nurse managers. Participants work across specialties like Obstetrics and Gynecology (21.5%), Pediatrics (16.0%), Rehabilitation (14.6%), Mental Health and Inpatient Services (both 13.9%), Critical Care (12.5%), and Admin/Education/Quality (7.6%). *The* Table 1 provides the demographic and professional characteristics of the 144 participants.

**Table 1:** Participant characteristic

Variable	Level	Value
N		144
Age in years, mean (SD)		51.7 (161.6)
Gender	Female	107 (74.3%)
	Male	37 (25.7%)
Marital Status	Married	139 (96.5%)
	Single	5 (3.5%)
Years of experience in HMC	1–5 yrs.	33 (22.9%)
	5-10yrs	50 (34.7%)
	11 and above yrs.	61 (42.4%)
Educational qualification	BSN/Diploma	117 (81.3%)
	Master’s degree or above	27 (18.8%)
Job Position	Charge Nurse	26 (18.2%)
	Nurse manager	13 (9.1%)
	Graduate Registered Nurse	87 (60.8%)
	Nurse educator	17 (11.9%)
Area of practice	Admin/Education/quality	11 (7.6%)
	Critical care	18 (12.5%)
	Inpatient	20 (13.9%)
	Mental health services	20 (13.9%)
	Obstetrics and Gynecology	31 (21.5%)
	Pediatrics	23 (16.0%)
	Rehabilitation	21 (14.6%)

### Assertiveness, empowerment, and self-esteem and their correlation

The mean score for assertiveness is 67.1 **±** 10.9, indicating moderate assertiveness with some variability among participants. Empowerment shows a mean score of 51.1 **± 5.9**, suggesting a generally moderate level of empowerment with less variability. Self-esteem has a mean score of 27.1 **±** 2.9, reflecting relatively high and consistent levels of self-esteem across the participants. Table 2 presents the descriptive statistics for assertiveness, empowerment, and self-esteem among the 144 participants.

**Table 2:** Descriptives for assertiveness, empowerment, and Self-Esteem

Variables	Value
N	144
Assertiveness, mean (SD)	67.1 (10.9)
Empowerment, mean (SD)	51.1 (5.9)
Self-esteem, mean (SD)	27.1 (2.9)

The mediation analysis using the structural equation model (SEM) reveals the relationships between empowerment, self-esteem, and assertiveness. The direct effect of self-esteem on assertiveness is not significant (β = -0.096, 95% CI: -0.258 to 0.065, *p* = 0.242), indicating no strong direct influence of self-esteem on assertiveness. Similarly, the effect of empowerment on self-esteem is also non-significant (β = 0.084, 95% CI: -0.0756 to 0.244, *p* = 0.302), suggesting empowerment does not significantly influence self-esteem. However, the direct effect of empowerment on assertiveness is significant (β = 0.207, 95% CI: 0.052 to 0.362, *p* = 0.009), showing that higher empowerment directly increases assertiveness. The mediation pathway (empowerment → self-esteem → assertiveness) is not significant (β = -0.004, 95% CI: -0.0156 to 0.007, *p* = 0.441), indicating that self-esteem does not mediate the relationship between empowerment and assertiveness. The overall model fit is excellent, as evidenced by the fit indices: CFI = 1.000, TLI = 1.000, RMSEA **= 0.000** with a 90% CI of [0.000, 0.000], SRMR = 0.000, and a non-significant chi-square statistic (χ² = 0.00), indicating the model fits the data perfectly. Table 3 presents mediation analysis of empowerment, self-esteem, and assertiveness using SEM model.

**Table 3:** Mediation analysis of empowerment, Self-Esteem, and assertiveness using SEM model

	Coefficient (95%CI)	*p* value
Self-esteem-> Assertiveness	-0.096(-0.258, 0.065)	0.242
Empower-> Self esteem	0.084(-0.0756,0.244)	0.302
Empower-> Assertiveness	0.207 (0.052, 0.362)	0.009
Empower-> Self-esteem-> Assertiveness	− 0.004(-0.0156, 0.007)	0.441

### Assertiveness, empowerment, and Self-Esteem across demographic variables

Independent t-tests and one-way ANOVA were conducted to examine the relationships between demographic and professional variables and the levels of assertiveness, empowerment, and self-esteem. For self-esteem, significant differences were observed across educational levels, years of experience, and job positions. Participants with a BSN/Diploma had slightly higher self-esteem 27.3 ± 3.0 compared to those with a master’s degree or above 26.1 ± 2.6; *p* = 0.053. Regarding years of experience, those with 1–5 years reported the highest self-esteem 28.3 ± 3.9 followed by those with 11 or more years 27.0 ± 2.9, and the lowest self-esteem was observed in participants with 5–10 years of experience 26.5 ± 2.0; *p* = 0.022. Among job positions, GRNs had the highest self-esteem mean = 27.7 ± 3.1, while nurse managers reported the lowest 25.5 ± 2.7. Charge nurses 26.0 ± 2.6 and nurse educators 26.8 ± 2.2 fell in between, with a significant p-value of 0.012.

No significant differences were found for assertiveness or empowerment across demographic and professional variables. Table 4*shows assertiveness*,* empowerment*,* and self-esteem across different demographic variables.*

**Table 4:** Demographic, professional characteristics, and their association with assertiveness, empowerment, and Self-Esteem

Variables	*N*	Assertiveness, mean ± SD	Empowerment, mean ± SD	Self-esteem, mean ± SD
**Gender**				
Female	107	66.5 ± 10.1	50.7 ± 5.6	27.1 ± 2.9
Male	37	68.9 ± 12.9	52.4 ± 6.5	27.1 ± 2.9
p-value		0.26	0.12	0.93
**Marital Status**				
Married	139	67.1 ± 11.0	51.1 ± 5.9	27.2 ± 2.9
Single	5	68.0 ± 8.9	51.2 ± 7.2	25.6 ± 3.0
p-value		0.86	0.98	0.25
**Education**				
BSN/Diploma	117	66.9 ± 10.9	51.4 ± 5.8	27.3 ± 3.0
Master’s degree or above	27	68.0 ± 11.2	50.1 ± 6.4	26.1 ± 2.6
p-value		0.66	0.32	0.053
**Year of experience**				
1–5 yrs.	33	66.7 ± 11.5	51.9 ± 6.8	28.3 ± 3.9
5-10yrs	50	68.3 ± 10.1	51.3 ± 6.1	26.5 ± 2.0
11 and above yrs.	61	66.4 ± 11.3	50.6 ± 5.2	27.0 ± 2.9
p-value		0.64	0.59	0.022
**Job Position**				
Charge Nurse	26	66.8 ± 12.1	50.5 ± 5.1	26.0 ± 2.6
Nurse manager	13	68.5 ± 11.8	51.1 ± 6.0	25.5 ± 2.7
GRN	87	66.5 ± 10.2	51.7 ± 6.3	27.7 ± 3.1
Nurse educator	17	69.8 ± 12.4	49.2 ± 4.6	26.8 ± 2.2
p-value		0.69	0.40	0.012
**Area of Practice**				
Admin/Education/quality	11	65.5 ± 12.0	48.3 ± 5.8	26.7 ± 2.5
critical care	18	68.9 ± 12.4	51.1 ± 6.4	26.7 ± 2.7
Inpatient	20	68.2 ± 12.3	51.0 ± 6.6	27.0 ± 3.0
Mental health services	20	70.8 ± 10.8	52.5 ± 5.9	26.5 ± 2.7
Obstetrics and Gynecology	31	64.7 ± 8.4	50.9 ± 6.0	27.8 ± 3.1
Pediatrics	23	63.2 ± 10.3	51.4 ± 5.2	27.1 ± 2.3
Rehabilitation	21	69.9 ± 10.8	51.6 ± 5.7	27.2 ± 3.9
p-value		0.17	0.69	0.78

## Discussion

The study reveals significant insights into the levels of assertiveness, empowerment, and self-esteem among nurses in Qatar, shedding light on the dynamic interplay between these psychological constructs and their sociodemographic determinants. This discussion critically examines the findings in the context of existing literature.

### Self-esteem

The relatively high self-esteem levels (27.1 ± 2.9) with previous studies, observed among nurses align with the resilience and confidence often associated with their roles [21]. High self-esteem is a protective psychological factor that helps nurses maintain a positive self-concept, even in high-stress environments. According to Rosenberg’s Self-Esteem Theory, individuals with higher self-esteem are more likely to exhibit adaptive behaviors and maintain emotional stability, traits essential for effective nursing practice [15].

Literature underscores the significance of self-esteem in fostering professional success and personal well-being among nurses. For instance, studies have linked high self-esteem with better coping mechanisms, reduced anxiety, and increased job satisfaction [22]. The findings suggest that nurses may benefit from workplace cultures and interventions that further enhance their self-esteem, ensuring sustained resilience in the face of occupational challenges [23].

### Empowerment

Empowerment levels in the study (51.1 ± 5.9) indicate that nurses experience relatively moderate empowerment compared with previous studies [24], suggesting the presence of structural or psychological barriers limiting their full professional autonomy. Empowerment is a multifaceted construct involving access to resources, decision-making authority, and a sense of control over one’s work environment [25]. Moderate scores suggest that while nurses may experience some degree of autonomy, systemic constraints, such as rigid hierarchies or limited participatory governance, may hinder them from fully exercising their professional potential [26].

Azizi et al. (2020) highlighted similar concerns, noting that empowerment in nursing is closely linked to job satisfaction, performance, and retention [5]. Their findings emphasize the role of organizational policies in fostering environments where nurses can engage in shared decision-making and access professional development opportunities. Addressing these barriers through targeted interventions, such as leadership training or participative management structures, could significantly enhance empowerment levels [27].

### Assertiveness

The study indicates a mean assertiveness score (67.1 ± 10.9), reflecting moderate assertiveness levels among nurses [28]. Assertiveness in nursing is critical for effective communication, patient advocacy, and navigating professional hierarchies. However, moderate levels suggest that while nurses demonstrate some assertive behaviors, challenges persist in asserting themselves in situations involving hierarchical dynamics or conflict resolution [29].

Previous literature corroborates these findings. Suzuki et al.(2021)identified that non-assertive behaviors in nurses are often linked to increased workplace stress and susceptibility to burnout [30]. Similarly, Zhou et al.(2024) emphasized that assertiveness training positively impacts job satisfaction and stress management, highlighting the need for interventions to strengthen assertiveness skills in nursing professionals [31]. The moderate levels observed in this study suggest a potential gap in training or support systems to cultivate assertiveness fully. Addressing these gaps could enhance interpersonal relationships, improve patient outcomes, and reduce role-related conflicts.

### Interrelationships among assertiveness, Self-Esteem, and empowerment

The interplay between assertiveness, self-esteem, and empowerment is crucial in understanding the psychological and professional experiences of nurses. High self-esteem likely bolsters confidence and contributes to moderate assertiveness levels, yet the structural limitations reflected in empowerment scores may constrain the application of these traits in practice [32]. For instance, a nurse with high self-esteem might be capable of assertive communication but may feel restricted by organizational policies or hierarchical dynamics, impacting their sense of empowerment [33].

Existing literature supports this interconnectedness. For example, Spreitzer (1996) proposed that empowerment is both a precursor to and an outcome of self-esteem and assertiveness [17]. Nurses who perceive themselves as empowered are more likely to advocate effectively for patients and themselves, fostering a positive feedback loop that enhances self-esteem and assertiveness [34]. Conversely, environments lacking empowerment can inhibit these traits, leading to professional dissatisfaction and reduced performance.

### Mediating effect among the study variables

The relationships between self-esteem, empowerment, and assertiveness provide valuable insights into the dynamics of these constructs. Empowerment showed a significant direct effect on assertiveness (β = 0.207, *p* = 0.009), indicating that nurses who feel empowered in their roles are more likely to exhibit assertive behaviors. This finding is consistent with Zimmerman’s Empowerment Theory (1995), which posits that autonomy, access to resources, and participatory decision-making are critical factors in enhancing motivation and assertiveness [35]. Studies by Duff (2019) also support the notion that empowered nurses are better equipped to navigate workplace challenges and advocate for their patients effectively [36].

Conversely, self-esteem did not have a significant direct effect on assertiveness (β = -0.096, *p* = 0.242). This contrasts with prior research, such as Ayhan & Seki Öz (2021), which identified strong correlations between self-esteem and assertive behaviors [37]. This discrepancy may be context-specific, reflecting differences in cultural norms, organizational structures, or the unique challenges of the Qatari healthcare system. While some studies suggest that sociocultural factors can affect assertiveness even in individuals with high self-esteem [3, 7] overall literature supporting this view remains limited.

Furthermore, the mediating role of self-esteem between empowerment and assertiveness was also non-significant (β = -0.004, *p* = 0.441). This suggests that while self-esteem and empowerment independently contribute to workplace well-being, their interplay does not significantly influence assertiveness in this population. This finding aligns with Hanson et al. (2020), who emphasized the direct impact of empowerment strategies on workplace behaviors, independent of self-esteem levels [38]. Given that the structural equation model (SEM) did not support the hypothesized mediation, we have critically considered potential reasons for the null findings. Specifically, the relatively small sample size (*n* = 144), while within acceptable SEM guidelines, may have limited the power to detect small or indirect effects. Additionally, measurement model limitations such as the use of observed variables (rather than latent constructs) could have contributed to attenuation of path coefficients.

The lack of a significant relationship between empowerment and self-esteem (β = 0.084, *p* = 0.302) further highlights the distinct nature of these constructs. While empowerment focuses on external and structural factors, such as autonomy and resource access, self-esteem is inherently internal, reflecting individual perceptions of self-worth. Previous studies, including Wang et al. (2013), have demonstrated that empowerment initiatives can enhance self-esteem over time, but this relationship may not be immediately evident in cross-sectional analyses [39].

### Associations with demographic variables

Significant associations were observed between self-esteem and certain demographic variables, while assertiveness and empowerment did not vary significantly across these characteristics. Nurses with BSN/Diplomas exhibited slightly higher self-esteem compared to those with advanced degrees (*p* = 0.053). This may reflect differences in role expectations, as nurses with advanced degrees often face greater professional demands and pressures, potentially impacting their self-esteem. These findings echo meta-analysis by Krauss & Orth (2022)., highlighted the role of workplace stressors in shaping self-esteem among nurses with differing educational qualifications [40].

Years of experience also influenced self-esteem, with nurses in their first 1–5 years of practice reporting the highest levels (*p* = 0.022). This finding may be attributed to the optimism and enthusiasm of early-career professionals [41]. However, a dip in self-esteem among those with 5–10 years of experience may signify the transitional challenges and increasing responsibilities faced during mid-career stages. Interestingly, self-esteem appeared to stabilize among nurses with over 11 years of experience, indicating that professional maturity and established routines may help mitigate earlier challenges.

Job positions significantly influenced self-esteem, with General Registered Nurses (GRNs) reporting the highest levels (mean = 27.7, SD = 3.1) and nurse managers the lowest (mean = 25.5, SD = 2.7; *p* = 0.012). These findings align with Duff’s (2019) research, which noted that leadership roles often come with added stress and responsibility, potentially leading to diminished self-esteem [42]. The unique stressors faced by nurse managers underscore the need for targeted interventions to support their psychological well-being and leadership capabilities [43].

## Limitation

This study has several limitations that should be acknowledged. Firstly, the design provides a snapshot of self-esteem, assertiveness, and empowerment levels, limiting the ability to infer causality or explore changes over time. Longitudinal studies would be more suited to examine the dynamic relationships among these variables and their evolution throughout nurses’ careers. Secondly, the reliance on self-reported measures with the absence of triangulation methods such as observational data, introduces the potential for social desirability bias, as participants might have overstated or understated their responses to align with perceived expectations. Additionally, the study focuses solely on nurses employed in facilities under Hamad Medical Corporation (HMC) in Qatar, which may limit the generalizability of findings to other healthcare settings or regions with different cultural and organizational contexts. Non-clinical and temporary nursing staff were excluded from the study to ensure role consistency among participants, as the focus was on nurses directly engaged in patient care. While this approach supported analytical clarity, it may limit the applicability of findings to those in more precarious or administrative roles. Lastly, while the sample size was adequate, a larger and more diverse sample might enhance the robustness of the findings and allow for more detailed subgroup analyses. Addressing these limitations in future research could provide deeper insights into the psychological well-being and professional growth of nurses.

## Implications and recommendations

The findings from this study highlight the pressing need for healthcare institutions to develop structured initiatives that support nurses’ psychological development, particularly in the areas of empowerment and assertiveness. Although the participating nurses demonstrated relatively high levels of self-esteem, the moderate levels of empowerment and assertiveness point to potential barriers within clinical environments that may limit their full professional expression. Assertiveness, being vital to effective communication, patient advocacy, and team collaboration, requires dedicated training that is both practical and context sensitive. Programs should move beyond generic instruction to incorporate interactive and experiential elements such as scenario-based learning, role-playing, guided reflection, and coaching. Moreover, integrating assertiveness content into orientation programs, continuing education curricula, and leadership development initiatives can help normalize such behaviors as essential professional competencies rather than optional soft skills.

On the institutional level, there is a clear opportunity to foster a culture of psychological empowerment through intentional policy reforms and organizational practices. This includes promoting participatory decision-making structures where nurses are actively involved in shaping clinical workflows and institutional policies. Empowerment can also be strengthened by establishing mentorship frameworks in which experienced nurses support less-experienced colleagues in building autonomy and navigating workplace challenges. Leadership styles that are inclusive and responsive to frontline feedback can further reinforce empowerment by validating the perspectives and contributions of nursing staff. In addition, formal recognition programs that acknowledge assertive communication and empowered behaviors may help cultivate these traits across the workforce. For such strategies to be effective, they must be embedded in broader institutional values that prioritize staff well-being, professional growth, and psychological safety. Future research should examine the long-term impact of these interventions, providing empirical guidance on how to sustain and scale successful empowerment and assertiveness development models across diverse healthcare settings.

## Conclusion

This study provides valuable insights into the self-esteem, assertiveness, and empowerment levels among nurses in Qatar, highlighting their critical roles in professional performance and psychological well-being. The findings reveal that while nurses exhibit moderate levels of assertiveness and empowerment, self-esteem remains relatively high, with significant variations across demographic and professional variables. The direct impact of empowerment on assertiveness underscores the importance of fostering supportive work environments that promote autonomy and decision-making. However, the lack of mediation through self-esteem suggests that contextual and structural factors play a more prominent role in shaping assertive behaviors within this cohort.

The study emphasizes the need for targeted interventions such as assertiveness training, empowerment programs, and self-esteem enhancement initiatives to address identified gaps and support nurses’ professional growth. Furthermore, the findings underscore the importance of aligning such programs with cultural and organizational contexts to maximize their effectiveness.

## Data Availability

The data that support the findings of this study are available from the corresponding author upon reasonable request.
